# Ongoing shielding behavior one year post COVID-19: results from a longitudinal study of patients with inflammatory arthritis

**DOI:** 10.1007/s00296-023-05430-2

**Published:** 2023-09-11

**Authors:** Melissa Sweeney, Lewis Carpenter, Savia de Souza, Hema Chaplin, Hsiu Tung, Emma Caton, James Galloway, Andrew Cope, Mark Yates, Elena Nikiphorou, Sam Norton

**Affiliations:** 1 Health Psychology Section, Health Psychology Department, Institute of Psychiatry, Psychology and Neuroscience, King’s College London, Guy’s Hospital, 5thFloor, Bermondsey Wing, Great Maze Pond, London, UK; 2 Centre for Rheumatic Diseases, King’s College London, London, UK

**Keywords:** Inflammatory arthritis, COVID-19, Shielding, Mental health

## Abstract

Many patients with inflammatory arthritis (IA) were instructed to shield during the COVID-19 pandemic. Despite the ending of lockdowns and vaccination, large proportions of IA patients were continuing to shield when it is no longer needed. Given the detrimental effects of shielding on mental and physical health, understanding the rates and reasons for shielding is needed to help clinicians advise patients accordingly. This study was a 12-month prospective study following participants with IA during the COVID-19 pandemic. The proportions of IA patients shielding at each time point were calculated. Additionally, regressions and odds ratios for shielding were determined to assess medication type, mental health, and risk perception. While the extent of shielding fluctuated over the year of lockdowns, nearly all IA patients (93.5%) were still engaging in some shielding in 2021, with nearly half (43%) still shielding most or all of the time. Medications that were previously considered higher risk were not significantly associated with higher rates of shielding (OR = 1.60, *p* = 0.29), but greater symptoms of depression in June 2020 (OR = 1.07, *p* = 0.03) was both associated with increased the odds of shielding in June 2021. The high rates of IA patients continuing to shield in 2021 put more strain on patients and professionals as social isolation is linked with worsening mental and physical health, as well as greater difficulty with self-management. It is important for clinicians to be aware of this trend to ease the stress on patients.

## Introduction

During the COVID-19 pandemic, many patients with inflammatory arthritis (IA) had increased risk of infection and were instructed to shield according to varying levels of risk [1]. Prior to this clarification, the UK government had issued guidance for anyone on immunosuppressant medications to self-isolate [2]. This likely led to increased health anxiety for IA patients.

Despite the ending of lockdowns and rollout of vaccinations, some IA patients continued shielding to differing extents [3]. This trend is concerning because shielding is associated with worse mental and physical health outcomes. In the IA population, those who were socially isolating were found to have higher rates of anxiety and depression compared with those who were not isolating [4].

In addition to the psychological impact of shielding, there are negative impacts on physical health. People do less physical activity and eat less healthy diets during social isolation, which are key parts of self-management for IA patients [5–7]. Additionally, studies with IA patients have shown that worse mental health is associated with worse physical health symptoms over time [8–10]. Thus, shielding may contribute to a negative feedback cycle between worsening mental and physical health. While shielding can reduce the risk of catching COVID-19, these costs of continued shielding may outweigh the benefits.

As IA patients have higher rates of mental health disorders than the general population, the additional stressor of social isolation could exacerbate pre-existing distress or risk for it [11]. An accurate picture of the rates of ongoing shielding and underlying causes would help to understand the need for clarification of risk for patients.

The aims of this study are twofold. First, it will determine the rates of shielding and the extent of social isolation that was ongoing in mid-2021. Second, it will examine potential factors that may be linked with higher rates of shielding, including medication type, mental health, and risk perception.

## Methods

### Design and recruitment

Data are from the IA-COVID cohort study, which followed patients with inflammatory arthritis longitudinally over one year from June 2020. It was comprised of a series of five waves of online questionnaires completed approximately every 3 months. The study began in June 2020 with follow-ups continuing until June 2021.

Participants were recruited for the study through social media or relevant charities. Eligibility criteria were: aged 18 years or older, living in the United Kingdom, and diagnosed with an IA condition. Three respondents were included from crown dependencies that form part of the British Isles but are not in the UK. Informed consent was obtained from all participants for the original IA-COVID study and related studies. Ethical approval was obtained from King’s College London Research Ethics Committee (LRS-19/20-18186). Subsamples of participants were included in a qualitative study and an ecological momentary assessment study [12].

### Measures

The questionnaires covered the following topics: IA condition, IA clinical care, self-management, disease outcomes, mental health, quality of life, COVID-19 clinical information, and COVID-19 experience. All of the questions were self-report. There were four waves of data collection: baseline (June 2020), September 2020, November 2020, and June 2021. The baseline and final data were used in these analyses.

### Arthritis condition

Participants self-reported information including: IA condition, diagnosis date, medication, and frequency. Symptom severity and quality of life measures were also obtained.

### Mental health measures

Depression was evaluated with the Personal Health Questionnaire Depression Scale (PHQ-8) at each timepoint. The PHQ-8 has been validated for various contexts [13].

### Shielding measures

Shielding was measured with the following researcher-designed question: “How much have you been social distancing or self-isolating by staying at home?” Responses included: “None of the time. I have continued my normal daily routine.”, “Some of the time. I have reduced some of the time I am in public spaces.”, “Most of the time. I leave only for essential journeys, such as food and doctor’s appointments.”, and “All of the time”.

Risk perception was measured with the following question: “How concerned do you feel about COVID-19?” Participants could choose five responses ranging from “Not at all concerned” to “Extremely concerned”.

The British Society for Rheumatology (BSR) risk stratification of patients with autoimmune rheumatic diseases was used to determine the risk category for shielding in response to COVID-19 according to disease treatment or other risk factors like age and comorbidities. Scores of 1 or less recommend social distancing, scores of 2 recommend self-isolation or social distancing at their discretion, and scores of 3 or more recommend shielding. Scores 2 and 3 were grouped together in this study due to the treatment information available from our questionnaire.

### Statistical analysis

The proportions of patients shielding were calculated at each timepoint between June 2020 and June 2021. These were further stratified by the strictness of the shielding according to the participants’ reports.

Ordinal logistic regression models were calculated for shielding behavior in June 2021, to determine the odds ratios for shielding by variables hypothesized to be potentially related to this decision. The variables considered were intensity of treatment (no disease modifying anti-rheumatic drug (DMARDs) treatments, conventional synthetic DMARDS, targeted DMARDs either alone or in combination with csDMARDS), the BSR risk stratification tool used to communicate need to shield based on current treatment, depressive and anxious symptoms at baseline, and risk perception at baseline*.* Each of the variables was tested in a separate model, adjusting for age and gender. Correlations between risk perception and depression and anxiety were also calculated. Finally, regression models were conducted to determine the association between depression and anxiety with shielding behavior. All analyses were conducted in STATA 17.0.

## Results

There were 338 participants included in the study sample. It was predominantly female (90.2%) with an average age of 47.9. Table 1 summarizes the participant demographic characteristics.

**Table 1:** Demographic and clinical characteristics

	Total sample
*N*	338
Age, Mean (SD)	47.89 (13.64)
Gender, Female %	90.2%
Education, % No formal qualifications O-level, GCSE or equivalent A-level or equivalent Undergraduate degree or equivalent Postgraduate degree or equivalent	3.55% 21.30% 21.01% 32.25% 21.89%
Depression, mean (SD)	10.53 (6.08)
Anxiety, mean (SD)	2.19 (1.98)
Risk perception, % Not at all concerned A little concerned Moderately concerned Very concerned Extremely concerned	1.84% 10.29% 26.47% 33.46% 27.94%
Treatment Intensity, % None NSAID Conventional DMARD Targeted DMARD	1.48% 26.92% 36.39% 35.21%
Risk stratification, % Social distance Self-isolate Shield	65.98% 29.29% 4.73%
Disease type, % Psoriatic arthritis Rheumatoid arthritis Spondyloarthritis Connective tissue disease Juvenile idiopathic arthritis	28.99% 29.59% 14.79% 25.15% 1.48%

### Rates of shielding at each timepoint

**Fig. 1:**
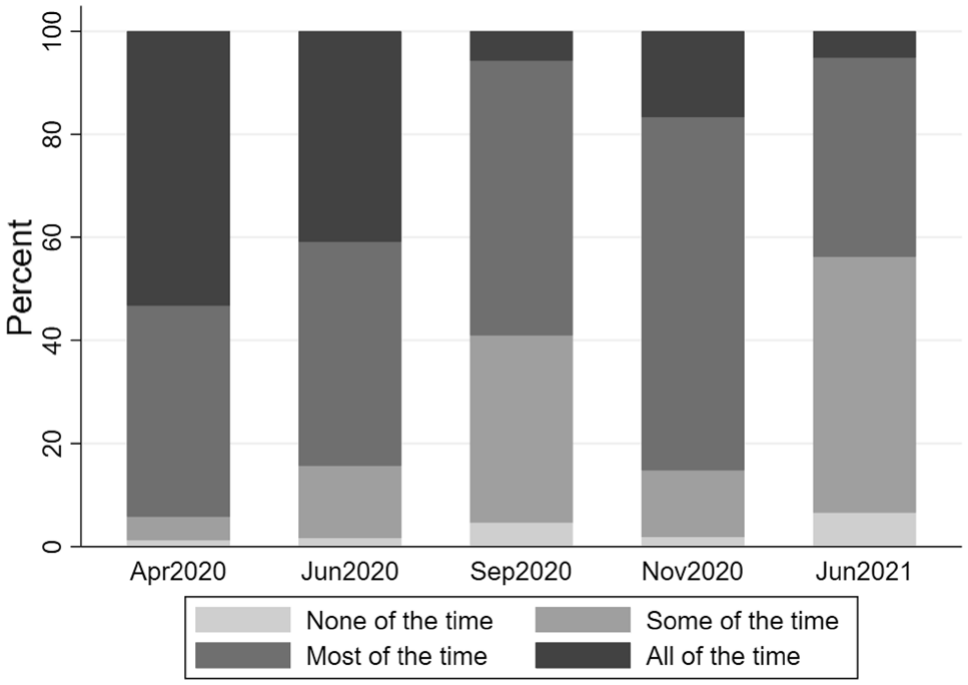
Proportions of IA patients shielding at each timepoint

The proportion of patients who are shielding to any extent decreased only slightly from the first lockdown, when 95.3% of patients were shielding, to 93.5% in June 2021 (Fig. 1). For those shielding, there was a large shift initially from shielding all of the time to most or some of the time by September 2020. After that point most people tended to remain either shielding most or some of the time shifting between these categories depending on the rates of COVID-19 infection in the population.

### Factors associated with continued shielding

Odds ratios for demographics showed no significant increase in shielding for age (OR = 1.02, *p* = 0.17) or gender (OR = 1.53, *p* = 0.54). Odds ratios for mental health did show increases in shielding for those with both higher symptoms of depression (OR = 1.07, *p* = 0.03) and higher symptoms of anxiety (OR = 1.21, *p* = 0.04). Risk perception also showed an increased risk of shielding (OR = 1.51, *p* = 0.03).

Odds ratios for treatment intensity found that those on a conventional DMARD were 1.76 times as likely to be shielding as those in the reference group (not on any DMARD). However, this increase was not significant (*p* = 0.17) (Table 2). Similarly, and paradoxically, those on targeted DMARDs were less likely to be shielding, (OR = 0.71) compared with those not on any DMARD, but the difference was also non-significant (*p* = 0.42).

**Table 2:** Odds ratio for shielding, adjusted for age and gender

	Odds ratio	95% CI	*p* value
Age	1.02	[0.99, 1.04]	0.17
Gender	1.53	[0.39, 6.05]	0.54
Depression	1.07	[1.01, 1.14]	**0.03**
Anxiety	1.21	[1.00, 1.46]	**0.04**
Risk perception	1.51	[1.05, 2.17]	**0.03**
Treatment Intensity Non-DMARD Conventional DMARD Targeted DMARD	1 1.76 0.71	– [0.78,3.96] [0.31,1.63]	– 0.17 0.42
BSR Risk stratification 0 = Social distancing 1 = Self-isolate 2 or 3 = Shield	1 0.78 0.79	– [0.38,1.61] [0.18, 3.37]	– 0.50 0.75

Finally, BSR risk stratification was used instead of treatment intensity. There appeared to be no significant increased risk of ongoing shielding for those with risk scores of 1 for self-isolation (OR = 0.78, *p* = 0.50) nor for scores of 2 or 3 for shielding (OR = 0.79, *p* = 0.75) compared with those with scores of 0, which corresponded with no recommendation to shield.

### Correlations

Finally, since mental health variables were the only significant predictors, these were further investigated. Table 3 shows correlations between mental health and other key variables.

**Table 3:** Correlations of predictors

	Depression	Anxiety	Shielding	Treatment intensity	Risk perception	Loneliness
Depression						
Anxiety	0.75*					
Shielding	0.16*	0.11				
Treatment intensity	0.33*	0.36*	0.30*			
Risk perception	− 0.02	< − 0.01	− 0.02	0.16*		
Loneliness	0.47*	0.51*	0.10	0.24*	< − 0.01	

In regression models of the final follow-up using mental health variables as outcomes, shielding was found to be associated with greater symptoms of depression [*b* = 2.71, 95% CI (1.27, 4.13), *p* < 0.01] and anxiety [b = 0.50, 95% CI (0.05, 0.95), *p* = 0.03] after controlling for age and gender. Thus, shielding behavior was associated with worse mental health. Whereas anxiety was more strongly related to future shielding behavior, greater shielding is associated with greater symptoms of depression.

## Discussion

This study found that, even one year after COVID-19, nearly all patients (93.5%) were still engaging in some level of shielding. Over half were still shielding at least most of the time if not all of the time in June 2021. It is important to be aware of ongoing shielding because various studies during COVID-19 found worse mental health associated with social isolation [14–16].

It is also important to note that in our analysis, shielding was not dependent on medication type, reflective of risk level, but solely psychological factors such as depression or risk perception. This knowledge can help clinicians address the shielding behavior by clarifying risk of COVID-19 for patients with inflammatory arthritis, particularly for those likely to have minimally increased risk of poor infectious outcome, including for those on immune-modifying drugs [17].

There is an overlap between depression and risk perception [18], which was also supported by our results. This is likely due to those with anxiousness and depressive symptoms having a more pessimistic outlook, expecting worse outcomes, which can lead to more worry about the risk of infection and higher risk perception. Since many patients have followed social distancing habits for over a year, there may be an initial hurdle of behavior change requiring some encouragement or guidance. It is important to keep in mind the negative feedback cycle wherein higher anxiety levels lead to greater shielding behavior, while more shielding is associated with greater depression.

Identifying patients who are continuing to shield when it is no longer needed can also help prevent a cycle of depression and social isolation and increased IA symptoms. As demonstrated in our previous paper, mental health can affect physical symptoms for IA patients even several months later so intervening can help decrease this cycle [19]. After the decline in mental and physical health that many IA patients endured over the last year, clarification about risk level may also help patients recover more easily by removing the excess burden of shielding.

This paper had limitations including self-report of all responses, which could affect accuracy. The frequency of change in government guidelines around social distancing regulations also contributed to difficulty accurately assessing shielding behavior as it could fluctuate with differing regulations. However, the study benefitted from a large sample size and range of IA conditions.

In conclusion, it is clear that mental health is a key aspect of continued shielding. Clinicians should be aware of ongoing consequences of shielding behavior to physical and mental health of IA patients. Screening for and providing mental health resources can lead to improved outcomes for patients.

## Data Availability

Data is available from authors upon request.
